# Amphiregulin Upregulation in Visfatin-Stimulated Colorectal Cancer Cells Reduces Sensitivity to 5-Fluororacil Cytotoxicity

**DOI:** 10.3390/biology13100821

**Published:** 2024-10-14

**Authors:** Wen-Shih Huang, Kuen-Lin Wu, Cheng-Nan Chen, Shun-Fu Chang, Ding-Yu Lee, Ko-Chao Lee

**Affiliations:** 1 Graduate Institute of Clinical Medical Sciences, College of Medicine, Chang Gung University, Taoyuan 333, Taiwan; wen1204@adm.cgmh.org.tw; 2 Division of Colon and Rectal Surgery, Department of Surgery, Chiayi Chang Gung Memorial Hospital, Chiayi 613, Taiwan; 3 Division of Colorectal Surgery, Department of Surgery, Kaohsiung Chang Gung Memorial Hospital, Kaohsiung Medical Center, Kaohsiung 833, Taiwan; kunn913@cgmh.org.tw; 4 Department of Biochemical Science and Technology, National Chiayi University, Chiayi 600, Taiwan; cnchen@mail.ncyu.edu.tw; 5 Department of Medical Research and Development, Chiayi Chang Gung Memorial Hospital, Chiayi 613, Taiwan; sfchang@cgmh.org.tw; 6 Center for General Education, Chiayi Chang Gung University of Science and Technology, Chiayi 613, Taiwan; 7 Department of Bioscience and Biotechnology, National Taiwan Ocean University, Keelung 202, Taiwan

**Keywords:** amphiregulin, colorectal cancer, drug resistance, 5-Fluororacil, visfatin

## Abstract

**Simple Summary:**

Abnormal elevation of AREG levels induced by Visfatin might trigger the development of CRC resistance to 5-FU therapy.

**Abstract:**

Colorectal cancer (CRC) has become a prevalent and deadly malignancy over the years. Drug resistance remains a major challenge in CRC treatment, significantly affecting patient survival rates. Obesity is a key risk factor for CRC development, and accumulating evidence indicates that increased secretion of adipokines, including Visfatin, under obese conditions contributes to the development of resistance in CRC to various therapeutic methods. Amphiregulin (AREG) is a member of the epidermal growth factor (EGF) family, which activates the EGF receptor (EGFR), influencing multiple tumorigenic characteristics of cancers. Abnormal expression levels of AREG in cancer cells have been associated with resistance to anti-EGFR therapy in patients. However, it remains unclear whether this abnormal expression also impacts CRC resistance to other chemotherapeutic drugs. The aim of this study is to examine whether AREG expression levels could be affected in CRC cells under Visfatin stimulation, thereby initiating the development of resistance to 5-fluororacil (5-FU). Through our results, we found that Visfatin indeed increases AREG expression, reducing the sensitivity of HCT-116 CRC cells to 5-FU cytotoxicity. Moreover, AREG upregulation is regulated by STAT3-CREB transcription factors activated by JNK1/2 and p38 signaling. This study highlights the significant role of AREG upregulation in CRC cells in initiating chemotherapeutic resistance to 5-FU under Visfatin stimulation. These findings provide a deeper understanding of drug resistance development in CRC under obese conditions and offer new insights into the correlation between an abnormal increase in AREG levels and the development of 5-FU-resistance in CRC cells, which should be considered in future clinical applications.

## 1. Introduction

Colorectal cancer (CRC) has become a malignant cancer with a high diagnosis rate and significant lethality over the years. Moreover, the age of patients diagnosed with CRC appears to have gradually declined in recent years [1,2]. The development of CRC is complex, involving various genetic and epigenetic modifications, including the accumulation of multiple gene mutations (e.g., APC, KRAS, and TP53), as well as the occurrence of microsatellite instability and CpG island methylator phenotypes [3]. Consequently, the therapeutic approach to CRC has been extensively investigated and developed, advancing significantly over the past few decades. Current treatments incorporate single and combined modalities of surgery, radiation, and chemotherapy. The main and highly effective drugs include 5-fluororacil (5-FU), capecitabine, oxaliplatin, and irinotecan. Additionally, targeted therapy drugs such as bevacizumab (an anti-vascular endothelial growth factor (VEGF) antibody) and cetuximab (an anti-epidermal growth factor receptor (EGFR) antibody) are also used based on the CRC’s unique molecular characteristics [4,5]. However, despite these efforts and advancements, challenges in the clinical management of CRC persist, affecting the effectiveness of treatments and cure rates for patients, primarily due to the development of drug resistance.

The mechanisms of drug resistance development in CRC can be multifaceted [6,7,8]. Enhancing the efficiency of the DNA repair system may mitigate the DNA damage caused by chemotherapeutic drugs, leading to reduced efficacy. Changes in the activity of membrane transporters may affect drug uptake and efflux, decreasing intracellular drug concentrations and their effectiveness. Additionally, modifications in proliferation and cell death signaling may result in abnormal cell growth and survival rates, contributing to resistance. Unusual activity and upregulation of EGF, EGFR, VEGF, and other angiogenic factors have also been proposed as causes of drug resistance development [6,7,8]. Amphiregulin (AREG), identified as a member of the EGF family, could contribute to its physiological and pathophysiological regulation in various cell types through the activation of EGFR [9,10,11]. AREG upregulation in the cancer microenvironment has been associated with the development of many types of cancers. This also suggests that AREG might be upregulated in different cell types, such as immune and vascular cells, within the cancer microenvironment to promote cancer growth [10,12]. Recently, accumulating evidence has indicated that AREG upregulation might contribute to drug resistance development in patients treated with the EGFR-targeted drug cetuximab [13]. However, its underlying mechanism and whether AREG upregulation could also cause resistance in patients treated with other chemotherapeutic drugs remain unclear.

Obesity has been established as a critical risk factor for at least 13 types of cancer, including CRC [14]. Moreover, evidence has suggested that this association is not limited to adults. Accumulating data have linked obesity in adolescence and early adulthood to an increased risk of developing cancer later in life [14]. Although more precise mechanisms should be further elucidated, our current understanding has already summarized several important initiators and signaling pathways linking obesity and carcinogenesis, such as chronic low-grade inflammation, oxidative stress, hyperinsulinemia, insulin resistance, and the secretion of cytokines and adipokines [14]. Adipokines, which can be secreted from adipose tissues/adipocytes and other tissues/cells, including cancers, have been crosslinked with the development of chronic low-grade inflammation in obesity, thereby promoting carcinogenesis and affecting the efficacy of chemotherapeutic drugs [15]. Recently, Visfatin, an adipokine also known as the extracellular form of nicotinamide phosphoribosyltransferase (eNAMPT), has been found to play an important role in CRC development due to the clinical positive correlation between its serum concentrations and CRC malignant stages [16,17,18,19]. Moreover, evidence has shown that Visfatin is able to decrease the sensitivity of CRC cells to chemotherapeutic drugs like capecitabine [20]. These findings highlight the great potential of Visfatin as a diagnostic molecular marker or therapeutic target, necessitating further examination of its mechanisms.

This study aimed to examine whether AREG levels could be affected in CRC cells under Visfatin stimulation and how this affects the sensitivity of CRC cells to the cytotoxicity of 5-FU. Our results demonstrated that Visfatin could upregulate AREG expression in CRC cells through the STAT3 and CREB transcription factors via JNK1/2 and p38 signaling. Consequently, this upregulation decreased the sensitivity of HCT-116 CRC cells to 5-FU cytotoxicity. These findings offer a more detailed understanding of how drug resistance develops in CRCs under Visfatin stimulation, providing new information that might influence future clinical applications. 

## 2. Materials and Methods

### 2.1. Materials

The materials for cell culture were sourced from Thermo (Waltham, MA, USA). We utilized several chemical inhibitors in this study, specifically PD98059 (P215, 25 μM), SP600125 (S5567, 20 μM), and SB203580 (559389, 10 μM), all obtained from Sigma (St. Louis, MO, USA). For antibody detection, the following primary antibodies were used, all procured from Cell Signaling Technology (Beverly, MA, USA): AREG (#42953, 1:800 dilution), phosphor-JNK1/2 (#4671, 1:1000 dilution), JNK1 (#3708, 1:1000 dilution), phosphor-p38 (#4511, 1:1000 dilution), p38 (#9212, 1:1000 dilution), phosphor-CREB (#9196, 1:500 dilution), CREB (#9197, 1:1000 dilution), phosphor-STAT3 (#9145, 1:1500 dilution), STAT3 (#9139, 1:1000 dilution), and β-actin (#3700, 1:2000 dilution). Specific siRNAs were employed for gene silencing, including control siRNA (AM4636), as well as siRNAs targeting AREG (ID 10236), CREB (ID 109994), and STAT3 (ID 116558), all obtained from Thermo (Waltham, MA, USA). Visfatin recombinant protein (130-09) was sourced from PeproTech (Rocky Hill, NJ, USA). All other materials utilized were also purchased from Sigma (St. Louis, MO, USA). 

### 2.2. Cell Culture

In this study, the HCT-116 human CRC cell line (60349) was obtained from the Bioresource Collection and Research Center (Hsinchu, Taiwan). The cells were seeded and cultured in Dulbecco’s modified Eagle medium, supplemented with 10% fetal bovine serum and 1% antibiotics, i.e., penicillin and streptomycin. All of the materials used in cell the culture were obtained from Thermo (Waltham, MA, USA). The cells were incubated in a 37 °C incubator supplemented with 5% carbon dioxide.

### 2.3. Real-Time PCR

HCT-116 CRC cells used for real-time PCR analysis were lysed using TRIzol solution. Following RNA purification, complementary DNA was synthesized from the purified mRNA using a reverse-transcription kit obtained from Thermo (Waltham, MA, USA). The expression patterns of AREG and GAPDG in the treated cells were analyzed and quantified using an ABI StepOnePlus machine (Applied Biosystems, Foster City, CA, USA). The reaction required complementary DNA, primers for AREG/GAPDH, and SYBR Green reagent (Applied Biosystems, Foster City, CA, USA). GAPDH expression pattern served as an internal control. The quantified equation was determined using the 2^−ΔΔ^*^C^*^t^ method. The primers used are AREG (+) 5′-ACCTACTCTGGGAAGCGTGA-3′ and AREG (−); 5′-AGCCAGGTA TTTGTGGTTCG-3′; GAPDH (+); 5′-CGGCGACGACCCATTCGAAC-3′; and GAPDH (−) and 5′-GAATCGAACCCTGATTCCCCGTC-3′. 

### 2.4. Western Blot

HCT-116 CRC cells used for Western blot analysis were lysed using a commercial cell lysis reagent (Millipore, Darmstadt, Germany), to which a cocktail solution was additionally added to inhibit the activity of protease and phosphatase (Roche, Basel, Switzerland). Following protein purification, the total protein concentration was quantified using a quantification kit (Bio-Rad, Hercules, CA, USA). For each sample, 50 μg of total proteins was loaded and run in the SDS-PAGE gel for electrophoresis. After transferring the proteins to the nitrocellulose membrane, the expression and phosphorylation patterns of the targeted proteins were analyzed using the indicated antibodies and a chemiluminescent detection system (Bio-Rad, Hercules, CA, USA). β-actin expression pattern served as an internal control.

### 2.5. MTT Assay

The cell viability/survival of HCT-116 CRC cells was assessed using the MTT (3-(4,5-dimethylthiazol-2-yl)-2,5-diphenyltetrazolium bromide) staining method. Following treatment for the specified durations, MTT solution at a concentration of 0.5 mg/mL was added to the culture medium in the dishes, and the cells were further incubated for an additional 3 h. After incubation, DMSO (dimethyl sulfoxide) was directly added to the culture medium in the dishes to dissolve the formazan crystals. Finally, the absorbance of each sample was measured at 570 nm.

### 2.6. Statistical Analysis

The results were derived from three independent experiments, all of which exhibited similar outcomes. Statistical analysis of the data from these three independent experiments was performed, and the results are presented as the mean ± the standard error of the mean (SEM). Comparisons between the two groups were conducted using an independent Student’s *t*-test, while analysis of variance (ANOVA) followed by Scheffe’s test was used for multiple comparisons. Statistical data were defined as significant if the *p*-value was smaller than 0.05. 

## 3. Results

### 3.1. Visfatin Upregulates AREG Expression in HCT-116 CRC Cells

**Figure 1:**
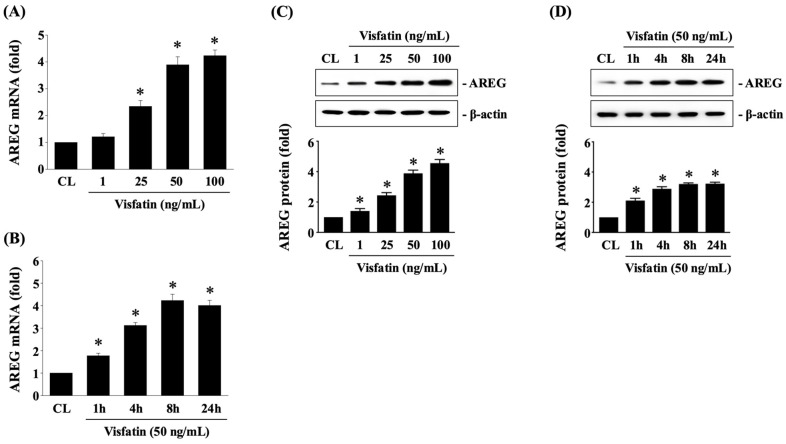
Visfatin upregulates AREG expression in HCT-116 CRC cells. HCT-116 CRC cells were either maintained as untreated controls or treated with Visfatin (1, 25, 50, and 100 ng/mL) for 1, 4, 8, and 24 h. The mRNA (**A**,**B**) and protein (**C**,**D**) expression of AREG was subsequently determined using real-time PCR and Western blot analysis, respectively. The data in (**A**–**D**) were calculated from the results of three independent experiments and are presented as the mean ± SEM. The results in (**C**,**D**) were obtained from three independent experiments, all of which exhibited similar outcomes. The results were considered statistically significant when *p* < 0.05. *, compared to the untreated control cells (CL).

The HCT-116 CRC cells were either maintained as untreated controls or treated with Visfatin at concentrations of 1, 25, 50, and 100 ng/mL for 1, 4, 8, and 24 h. The mRNA and protein expression of AREG was subsequently determined using real-time PCR and Western blot analysis, respectively. Treatment with Visfatin significantly upregulated both the mRNA (Figure 1A,B) and protein (Figure 1C,D and Figure S1) expression of AREG in a dose-dependent manner (observed at concentrations from 25 to 100 ng/mL) at the 8 h time point, as well as in a time-dependent manner (at 50 ng/mL) from 1 to 24 h compared to the untreated control cells.

### 3.2. Visfatin-Increased AREG Levels Attenuate the Cytotoxicity of HCT-116 CRC Cells in Response to 5-FU

**Figure 2:**
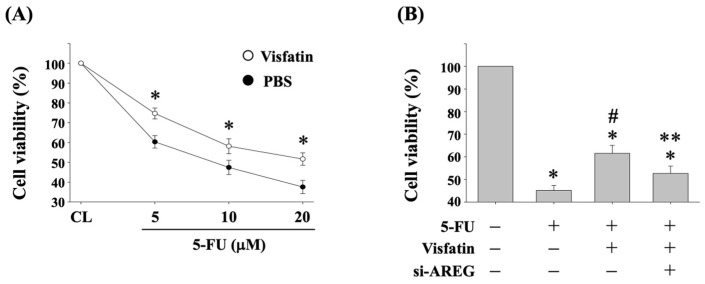
Visfatin-increased AREG levels attenuate the cytotoxicity of HCT-116 CRC cells in response to 5-FU. (**A**) HCT-116 CRC cells were pretreated with either vehicle (PBS) or Visfatin (50 ng/mL) for 1 h. They were then either maintained as controls or treated with 5-FU (5, 10, and 20 μM) for 24 h. (**B**) HCT-116 CRC cells were transfected with AREG-specific siRNA for 48 h. Subsequently, the cells were pretreated with PBS or Visfatin (50 ng/mL) for 1 h and then either maintained as controls or treated with 5-FU (10 μM) for 24 h. (**A**,**B**) The viability of treated cells was assessed using the MTT assay. The data were calculated from the results of three independent experiments and are presented as the mean ± SEM. The results were considered statistically significant when *p* < 0.05. *, compared to the untreated control cells (CL). #, compared to cells treated with 5-FU alone. **, compared to cells treated with Visfatin and 5-FU.

Previous studies have shown that AREG expression is associated with the development of drug resistance in CRC therapy [13]. To investigate if Visfatin-induced AREG upregulation affects the cytotoxicity of HCT-116 CRC cells to 5-FU, the cells were pretreated with either vehicle (PBS) or Visfatin (50 ng/mL) for 1 h. They were then either maintained as controls or treated with 5-FU at concentrations of 5, 10, and 20 μM) for 24 h. Cell viability was assessed using the MTT assay. The results indicated that cells pretreated with Visfatin exhibited reduced sensitivity to 5-FU, leading to a higher survival rate compared to those treated with PBS and 5-FU (Figure 2A). Furthermore, to knockdown endogenous gene expression, the cells were transfected with AREG-specific siRNA for 48 h. Following this, the cells were pretreated with PBS or Visfatin (50 ng/mL) for 1 h and subsequently either maintained as controls or treated with 5-FU (10 μM) for 24 h. Cell viability was again evaluated using the MTT assay. As expected, 5-FU treatment significantly reduced the cell survival rate compared to untreated control cells (Figure 2B). However, co-treatment with Visfatin and 5-FU mitigated this reduction in viability compared to cells treated with 5-FU alone (Figure 2B). Importantly, AREG gene knockdown restored the sensitivity of HCT-116 cells to 5-FU, reversing the attenuation caused by Visfatin compared to cells treated with Visfatin and 5-FU (Figure 2B).

### 3.3. JNK1/2 and p38 Signaling Regulate AREG Upregulation in HCT-116 CRC Cells under Visfatin Stimulation

**Figure 3:**
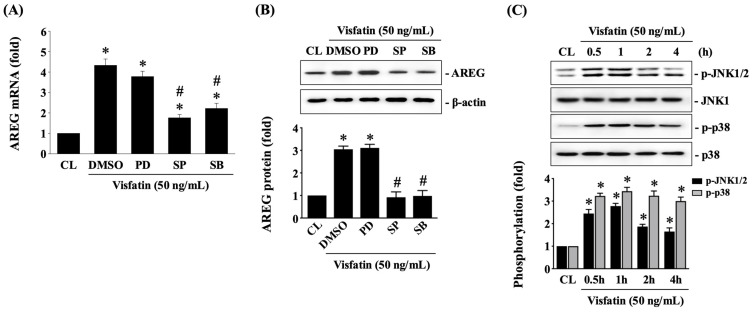
JNK1/2 and p38 signaling regulate AREG upregulation in HCT-116 CRC cells under Visfatin stimulation. (**A**,**B**) HCT-116 CRC cells were pretreated with DMSO (vehicle), PD98059 (an ERK1/2 inhibitor at 25 μM), SP600125 (a JNK1/2 inhibitor at 20 μM), or SB203580 (a p38 inhibitor at 10 μM) for 30 min and then either maintained as controls or treated with Visfatin (50 ng/mL) for 8 h. The mRNA (**A**) and protein (**B**) expression of AREG was determined using real-time PCR and Western blot analysis, respectively. (**C**) HCT-116 CRC cells were maintained as controls (CL) or treated with Visfatin (50 ng/mL) for 0.5, 1, 2, and 4 h, and then the phosphorylation of JNK1/2 and p38 kinases was examined using Western blot analysis. The data in (**A**–**C**) were calculated from the results of three independent experiments and are presented as the mean ± SEM. The results in (**B**,**C**) were obtained from three independent experiments, all of which exhibited similar outcomes. The results were considered statistically significant when *p* < 0.05. *, compared to the untreated control cells (CL). #, compared to cells treated with DMAO.

To further investigate whether MAPK signaling pathways, specifically ERK1/2, JNK1/2, and p38 kinases, regulate AREG upregulation in Visfatin-treated HCT-116 CRC cells, the cells were pretreated with DMSO (vehicle), PD98059 (an ERK1/2 inhibitor at 25 μM), SP600125 (a JNK1/2 inhibitor at 20 μM), or SB203580 (a p38 inhibitor at 10 μM) for 30 min. Following this pretreatment, the cells were either maintained as controls or treated with Visfatin (50 ng/mL) for 8 h. The mRNA and protein expression of AREG was then determined using real-time PCR and Western blot analysis, respectively. Pretreatment with SP600125 or SB203580 significantly reduced AREG mRNA (Figure 3A) and protein (Figure 3B and Figure S2) expression in Visfatin-treated cells compared to those treated with DMSO and Visfatin. In contrast, PD98059 pretreatment did not alter the effects of Visfatin on AREG expression. Additionally, treatment with Visfatin (50 ng/mL) significantly induced the phosphorylation of JNK1/2 and p38 kinases (Figure 3C and Figure S2), with phosphorylation levels peaking at 1 h. Although the phosphorylation levels declined thereafter, they remained elevated at 2 and 4 h post-stimulation compared to the untreated control cells.

### 3.4. CREB Affects AREG Upregulation and Subsequent 5-FU-Initiated Cytotoxicity in Visfatin-Stimulated HCT-116 CRC Cells

**Figure 4:**
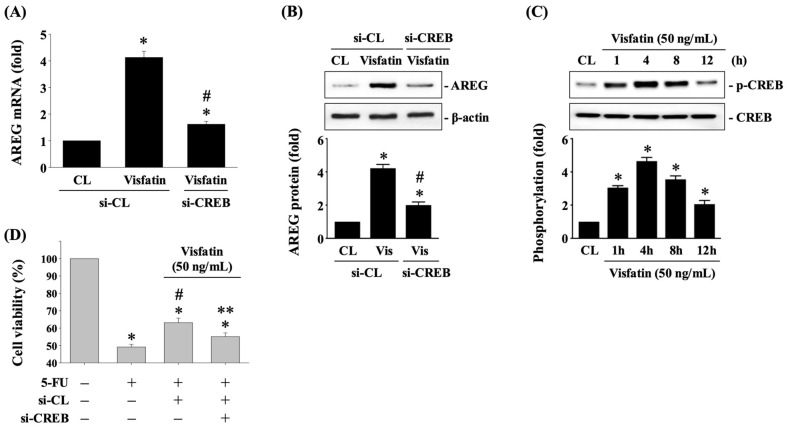
CREB affects AREG upregulation and subsequent 5-FU-initiated cytotoxicity in Visfatin-stimulated HCT-116 CRC cells. (**A**,**B**) HCT-116 CRC cells were transfected with control (si-CL)- or CREB (si-CREB)-specific siRNA for 48 h and then either maintained as controls or treated with Visfatin (50 ng/mL) for 8 h. The mRNA (**A**) and protein (**B**) expression of AREG was determined using real-time PCR and Western blot analysis, respectively. (**C**) The HCT-116 CRC cells were maintained as controls (CL) or treated with Visfatin (50 ng/mL) for 1, 4, 8, and 12 h, and then the phosphorylation of CREB was examined using Western blot analysis. (**D**) The HCT-116 CRC cells were transfected with control (si-CL)- or CREB (si-CREB)-specific siRNA for 48 h and then pretreated with vehicle (PBS) or Visfatin (50 ng/mL) for 1 h. Subsequently, the cells were either maintained as controls or treated with 5-FU (10 μM) for 24 h. The viability of treated cells was examined using the MTT assay. The data in (**A**–**D**) were calculated from the results of three independent experiments and are presented as the mean ± SEM. The results in (**B**,**C**) were obtained from three independent experiments, all of which exhibited similar outcomes. The results were considered statistically significant when *p* < 0.05. *, compared to (**A**,**B**) cells treated with si-CL-CL and (**C**,**D**) the untreated control cells. #, compared to (**A**,**B**) cells treated with si-CL and Visfatin and (**D**) cells treated with 5-FU alone. **, compared to (**D**) cells treated with si-CL, Visfatin, and 5-FU.

CREB is one of the transcription factors that controls AREG expression in a context-dependent pattern [11]. To investigate whether CREB regulates AREG expression in HCT-116 CRC cells in response to Visfatin, cells were transfected with either control siRNA or CREB-specific siRNA for 48 h. Following this, the cells were either maintained as controls or treated with Visfatin (50 ng/mL) for 8 h. The mRNA and protein expression of AREG was then determined using real-time PCR and Western blot analysis, respectively. Knockdown of CREB endogenous gene expression significantly reduced AREG mRNA (Figure 4A) and protein (Figure 4B and Figure S3) expression in Visfatin-stimulated cells compared to cells treated with control siRNA and Visfatin. Additionally, Visfatin treatment induced CREB phosphorylation in a time-dependent pattern, peaking at 4 h and then declining, yet remaining elevated at 8 and 12 h post-stimulation compared to the untreated control cells (Figure 4C and Figure S3). Moreover, knockdown of the CREB endogenous gene in Visfatin-treated HCT-116 CRC cells significantly recovered their sensitivity to 5-FU cytotoxicity, resulting in a marked reduction in cell survival compared to those treated with control siRNA, Visfatin, and 5-FU (Figure 4D).

### 3.5. STAT3 Affects AREG Upregulation and Subsequent 5-FU-Initiated Cytotoxicity in Visfatin-Stimulated HCT-116 CRC Cells

**Figure 5:**
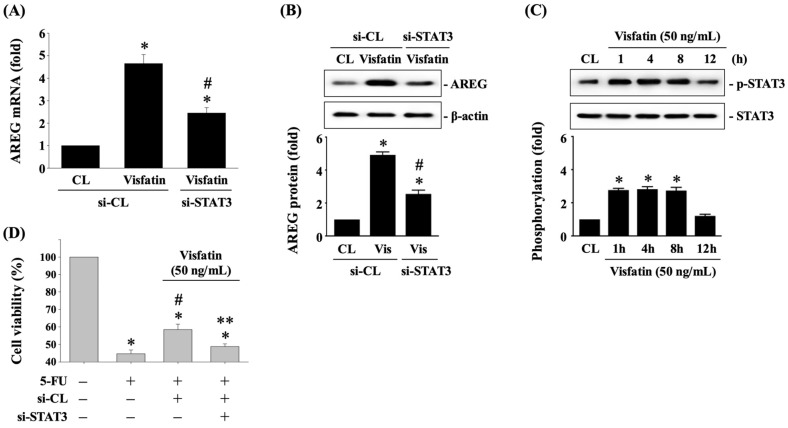
STAT3 affects AREG upregulation and subsequent 5-FU-initiated cytotoxicity in Visfatin-stimulated HCT-116 CRC cells. (**A**,**B**) HCT-116 CRC cells were transfected with control (si-CL)- or STAT3 (si-STAT3)-specific siRNA for 48 h and then either maintained as controls or treated with Visfatin (50 ng/mL) for 8 h. The mRNA (**A**) and protein (**B**) expression of AREG was determined using real-time PCR and Western blot analysis, respectively. (**C**) HCT-116 CRC cells were maintained as controls (CL) or treated with Visfatin (50 ng/mL) for 1, 4, 8, and 12 h and then the phosphorylation of STAT3 was examined sing Western blot analysis. (**D**) HCT-116 CRC cells were transfected with control (si-CL)- or STAT3 (si-STAT3)-specific siRNA for 48 h and then pretreated with vehicle (PBS) or Visfatin (50 ng/mL) for 1 h. Subsequently, the cells were either maintained as controls or treated with 5-FU (10 μM) for 24 h. The viability of treated cells was examined using the MTT assay. The data in (**A**–**D**) were calculated from the results of three independent experiments and are presented as the mean ± SEM. The results in (**B**,**C**) were obtained from three independent experiments, all of which exhibited similar outcomes. The results were considered statistically significant when *p* < 0.05. *, compared to (**A**,**B**) cells treated with si-CL-CL and (**C**,**D**) the untreated control cells. #, compared to (**A**,**B**) cells treated with si-CL and Visfatin and (**D**) the cells treated with 5-FU alone. **, compared to (**D**) cells treated with si-CL, Visfatin, and 5-FU.

STAT3 is another critical transcription factor involved in the regulation of AREG expression [21]. To investigate whether STAT3 regulates AREG expression in HCT-116 CRC cells in response to Visfatin, the cells were transfected with either control siRNA or STAT3-specific siRNA for 48 h. Following transfection, the cells were either maintained as controls or treated with Visfatin (50 ng/mL) for 8 h. The mRNA and protein expression of AREG were determined using real-time PCR and Western blot analysis, respectively. Knockdown of STAT3 endogenous gene expression significantly reduced AREG mRNA (Figure 5A) and protein (Figure 5B and Figure S4) expression in Visfatin-stimulated cells compared to cells treated with control siRNA and Visfatin. Additionally, treatment with Visfatin induced STAT3 phosphorylation in a time-dependent manner, peaking within 1 h and persisting up to 8 h and then declining to basal levels after 12 h of stimulation compared to untreated control cells (Figure 5C and Figure S4). Moreover, knockdown of the STAT3 endogenous gene in Visfatin-treated HCT-116 CRC cells significantly recovered their sensitivity to 5-FU cytotoxicity, resulting in a marked reduction in cell survival compared to those treated with control siRNA, Visfatin, and 5-FU (Figure 5D).

### 3.6. JNK1/2 and p38 Signaling Affects CREB and STAT3 Phosphorylation and Subsequent 5-FU-Initiated Cytotoxicity in Visfatin-Stimulated HCT-116 CRC Cells

**Figure 6:**
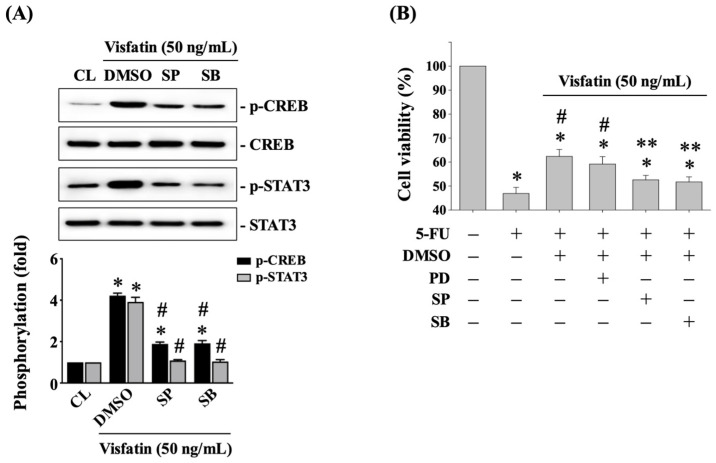
JNK1/2 and p38 signaling affects CREB and STAT3 phosphorylation and subsequent 5-FU-initiated cytotoxicity in Visfatin-stimulated HCT-116 CRC cells. (**A**) HCT-116 CRC cells were pretreated with DMSO (vehicle), SP600125 (a JNK1/2 inhibitor at 20 μM), or SB203580 (a p38 inhibitor at 10 μM) for 30 min and then either maintained as controls or treated with Visfatin (50 ng/mL) for 4 h. The phosphorylation of CREB and STAT3 was determined using Western blot analysis. (**B**) HCT-116 CRC cells were pretreated with DMSO (vehicle), PD98059 (an ERK1/2 inhibitor at 25 μM), SP600125 (a JNK1/2 inhibitor at 20 μM), or SB203580 (a p38 inhibitor at 10 μM) for 30 min and then treated with vehicle (PBS) or Visfatin (50 ng/mL) for 1 h. Subsequently, the cells were then either maintained as controls or treated with 5-FU (10 μM) for 24 h. The viability of the treated cells was examined using the MTT assay. The results in (**A**) were obtained from three independent experiments, all of which exhibited similar outcomes. The data in (**A**,**B**) were calculated from the results of three independent experiments and are presented as the mean ± SEM. The results were considered statistically significant when *p* < 0.05. *, compared to untreated control cells. #, compared to cells treated with (**A**) DMSO and Visfatin and (**B**) 5-FU alone. (**B**) **, compared to cells treated with DMSO and 5-FU.

Finally, HCT-116 CRC cells were pretreated with DMSO (vehicle), SP600125 (a JNK1/2 inhibitor at 20 μM), or SB203580 (a p38 inhibitor at 10 μM) for 30 min. Following this pretreatment, the cells were either maintained as controls or treated with Visfatin (50 ng/mL) for 4 h. The phosphorylation of CREB and STAT3 was then determined using Western blot analysis. Pretreatment with SP600125 or SB203580 significantly attenuated CREB and STAT3 phosphorylations in Visfatin-stimulated cells compared to cells treated with DMSO and Visfatin (Figure 6A and Figure S5). Additionally, pretreating cells with SP600125 or SB203580 significantly recovered the sensitivity of HCT-116 CRC cells to 5-FU cytotoxicity, resulting in a significant reduction in cell survival compared to those treated with DMSO, Visfatin, and 5-FU (Figure 6B). In contrast, pretreatment with PD98059 did not have this recovery effect (Figure 6B).

## 4. Discussion

Cancer is a complex and context-dependent disease, making clinical therapy challenging because each patient might undergo various genetic and epigenetic changes, even with the same type of cancer. Additionally, the development of resistance during treatment might further influence the efficacy of different therapeutic methods. Therefore, a more extensive and precise examination of the molecular mechanisms involved is always needed. The rationale of this study was to examine whether AREG expression could be regulated in CRC cells under obesity/adipokine situations, thereby initiating the development of resistance to chemotherapeutic drugs. The results showed that: (i) Visfatin indeed increases AREG expression in HCT-116 CRC cells, thereby reducing the sensitivity of the cells to 5-FU cytotoxicity; (ii) the potential transcription factors controlling AREG upregulation include STAT3 and CREB; and (iii) JNK1/2 and p38 signaling are important downstream pathways of Visfatin, influencing STAT3/CREB activation and subsequent AREG upregulation. 

NAMPT has been identified to have dual roles in regulating normal cell function and pathogenic development. Firstly, it serves as an essential intracellular enzyme contributing to nicotinamide dinucleotide (NAD) homeostasis, critical for ATP synthesis and energy supply. Secondly, in its extracellular form, known as Visfatin, it is involved in immune regulation and inflammation [15,22,23]. Accumulating evidence indicates that abnormal upregulation of both intracellular enzymatic activity and extracellular secretion is associated with the carcinogenesis of various cancers, including CRCs [15,23]. It has been further suggested that the increased enzymatic activity of NAMPT may result from the rapid proliferative rate and the shorter half-life of NAD in cancer cells [22]. Additionally, the increased Visfatin level in the cancer microenvironment has been found to be due to secretion from adipocytes, immune cells, and/or cancer cells themselves [22]. Thus, the secreted Visfatin is considered to have both paracrine and autocrine effects during carcinogenesis. Over the past decade, several clinical studies have already evidenced a positive correlation between serum Visfatin concentration and CRC development and stages [24,25,26,27]. Furthermore, studies by ourselves and others (including previous studies and the present study) show that Visfatin reduces the sensitivity of CRC cells to chemotherapeutic drugs, e.g., capecitabine and 5-FU [20,28,29]. This indicates that increased Visfatin secretion could be a primary cause of drug resistance development in CRCs. Considering these findings, the inhibition of NAMPT, including its enzymatic activity and Visfatin effects, has been suggested as a potential therapeutic target for cancer treatment. Actually, various NAMPT inhibitors have already been applied in clinical trials, but they have encountered different challenges [22,30]. However, due to the significant clinical and in vivo/in vitro evidence, blocking Visfatin’s effects remains a potential medical strategy for inhibiting cancer development and resistance induction in CRCs and even other cancer types.

AREG can exhibit duel-faced regulatory activity, maintaining normal physiology and promoting pathogenic disease development. Its different regulatory roles depend on its expression levels, the target cells, and the stimulation of cytokines, growth factors, or hormones [11,31]. The present results show that AREG expression could be upregulated in HCT-116 CRC cells stimulated by Visfatin, thereby affecting CRC cells’ sensitivity to 5-FU cytotoxicity. Our findings suggest that increased AREG expression in CRC cells might be a mechanism of drug resistance to 5-FU in a Visfatin-containing cancer microenvironment and under obese conditions. As a ligand of EGFR, AREG upregulation has been widely studied and linked to abnormal proliferation, migration, invasion, and anti-apoptosis characteristics in transformed cells [11,31]. However, clinical application of the EGFR-neutralizing drug cetuximab has shown that AREG expression/secretion levels can compensatorily increase in the patient’s cancer microenvironment, leading to drug resistance development and being a predictive marker for anti-EGFR treatment [11,13,32,33]. Additionally, in vitro studies have also found increased AREG expression/secretion in radiation-resistant pancreatic cancer cells and cisplatin-resistant breast and hepatoma cancer cells but not in cisplatin-stimulated lung cancer cells [11,31]. Summarizing these data and our findings, the unusual increases in AREG expression and secretion might not only be a potential initiator for the transformation of various cancers but also an important drug resistance mechanism in some cancers, reducing their sensitivity to radiotherapy or chemotherapeutic drugs. Notably, although anti-AREG therapy has been considered a possible therapeutic strategy in cancer treatment, the competing concept indicates that EGFR-targeting therapy might still be the predominant developing direction [11,31]. Thus, more extensive investigation and precise elucidation of the molecular mechanism of AREG overexpression in cancer cells, including CRCs, are needed to support its future clinical application. 

The expression of AREG can be controlled via transcriptional, post-transcriptional, and epigenetic regulation [11]. Our results showed that the Visfatin-induced increase in AREG levels is regulated by STAT3-CREB signaling. Several important transcription factors have been found to influence AREG transcription, including CREB, p53, WT1 (Wilms tumor suppressor), β-catenin, HIF-2 (hypoxia-inducible factor-2), and AP-1 (activated protein-1) [11]. This also indicates that the control of AREG expression is in a context-dependent manner, as regulation by these transcription factors occurs in different cell types under various stimuli. Among these transcription factors, CREB appears to be the most common. Studies have evidenced that CREB can control AREG upregulation in immune T cells, cancer cells, and oral epithelial cells under different situations [34,35,36,37]. These findings support our results regarding the role of CREB in AREG upregulation in Visfatin-stimulated CRC cells. Additionally, STAT3 is a versatile transcription factor that plays a critical role in the development of inflammation and carcinogenesis [38]. The transcriptional activity of STAT3 is primarily activated by JAK1/2 signaling and can also be influenced by MAPK signaling, i.e., ERK1/2, JNK1/2, and p38 [38]. Interestingly, in lung cancer cells, STAT3 transcriptional activity can be activated to promote Visfatin expression, thereby initiating the development of cisplatin resistance [39]. However, other studies have shown the opposite correlation between Visfatin and STAT3, finding that Visfatin could activate STAT3 to regulate the expression of target genes, leading to microenvironment inflammation and malignancy in various cells [40]. Our present results also demonstrate the downstream role of STAT3 in Visfatin-stimulated CEC cells, showing that its activation could further enhance CREB’s transcriptional regulation of AREG expression. However, our designs have limitations, as we did not further examine whether STAT3 directly contributes to AREG transcription and whether STAT3 and CREB could cooperatively control AREG transcription. 

Furthermore, some other limitations of this study should also be discussed. (i) Obesity is a multifaceted contributor to cancer resistance due to its complex regulatory mechanisms. While our findings emphasize the role of Visfatin stimulation, they do not fully elucidate the broader implications of obesity. The intricate correlation between obesity, elevated serum Visfatin concentration, and the development of 5-FU-resistant CRCs warrants further investigation using both animal models and clinical data. (ii) Additionally, the expression levels and/or mutations of EGFR across different CRC subtypes should be further examined. Specifically, their roles in resistance development, particularly through the abnormal upregulation of AREG, need to be investigated. Understanding EGFR’s involvement could provide insights into personalized treatment approaches for CRC patients in the future.

## 5. Conclusions

In conclusion, our results show that Visfatin is able to increase AREG expression through JNK1/2/p38 signaling and STAT3-CREB transcription factors in CRC cells, thereby reducing the sensitivity of CRC cells to 5-FU cytotoxicity. This study highlights the significant role of AREG upregulation in CRC cells in initiating chemotherapeutic resistance to 5-FU under Visfatin stimulation. These findings provide a deeper understanding of drug resistance development in CRC under obese conditions and offer new insights into the correlation between an abnormal increase in AREG levels and the development of 5-FU resistance in CRC cells, which should be considered in future clinical applications.

## Data Availability

The data that support the findings of the current study are available from the corresponding author upon reasonable request. The raw data of the Western blot results (Figure 1 and Figure 3, Figure 4, Figure 5 and Figure 6) are presented in Supplementary Materials Figure S1–S5.

## Supplementary Materials

The following supporting information can be downloaded at: https://www.mdpi.com/article/10.3390/biology13100821/s1. The raw data of the Western blot results (Figure 1, Figure 3, Figure 4, Figure 5 and Figure 6) are presented in Supplementary Materials Figure S1–S5.
