# Supplementary material for: Amphiregulin Upregulation in Visfatin-Stimulated Colorectal Cancer Cells Reduces Sensitivity to 5-Fluororacil Cytotoxicity
Source: Biology (Basel). 2024 Oct 14;13(10):821. doi: 10.3390/biology13100821 (PMC11505234; doi:10.3390/biology13100821)
Supplement: Supplementary file 1 [file biology-13-00821-s001.zip › biology-3224481-supplementary.pdf]

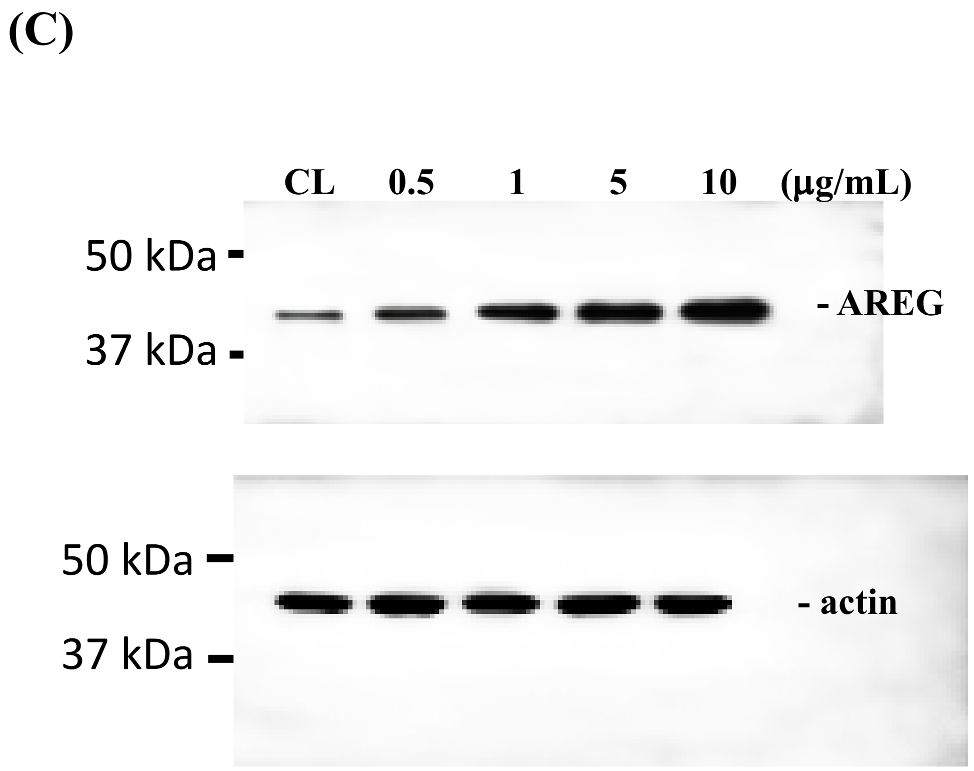

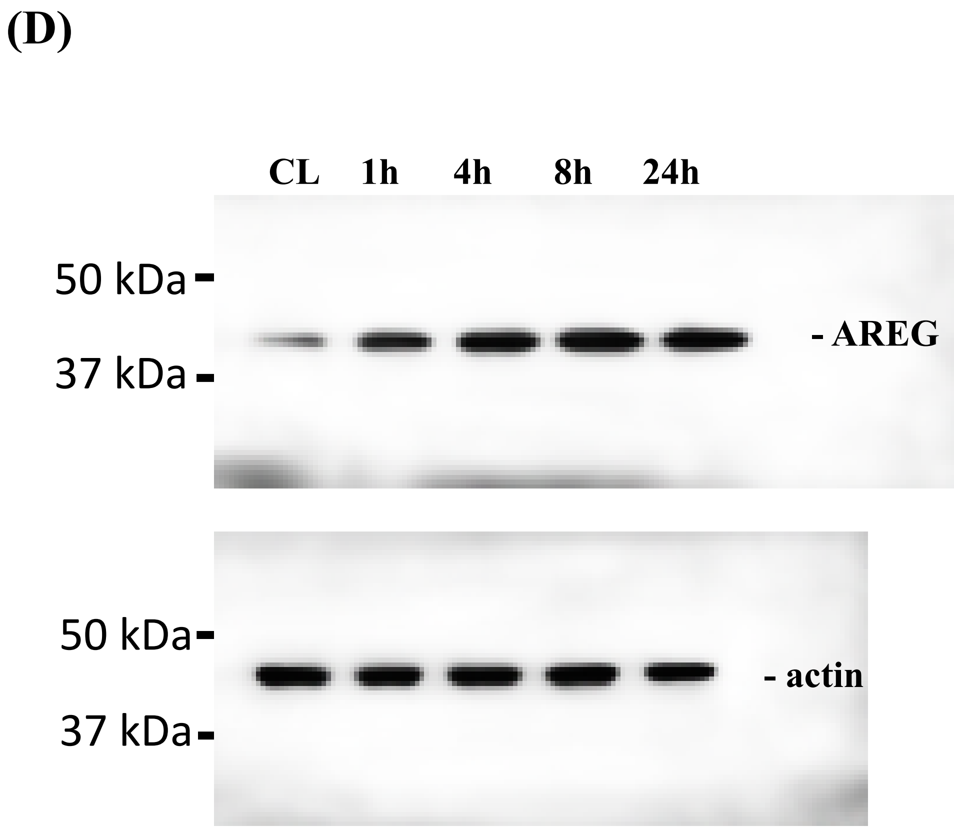

Figure S1. Raw data of Figure 1C and 1D

(B)

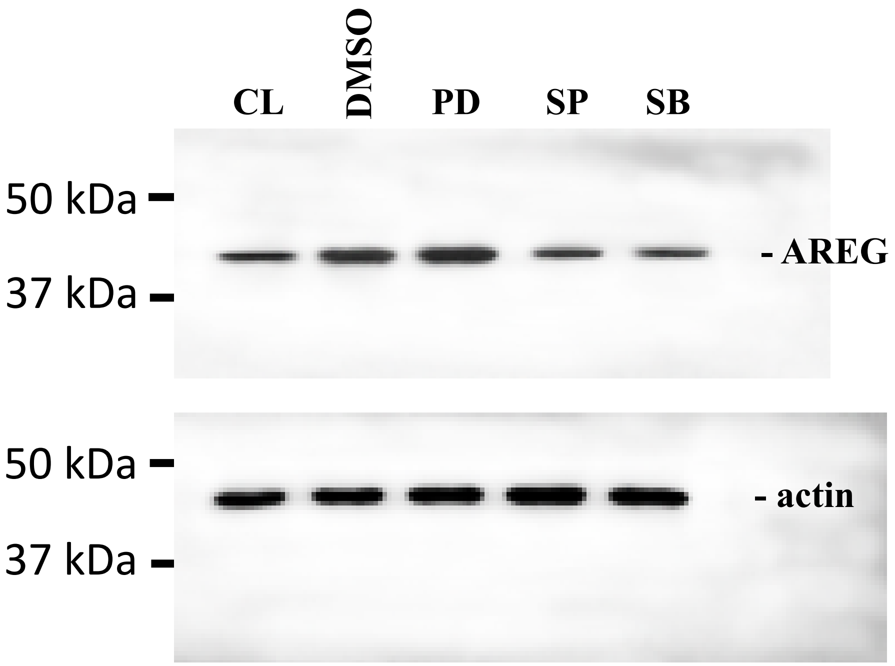

(C)

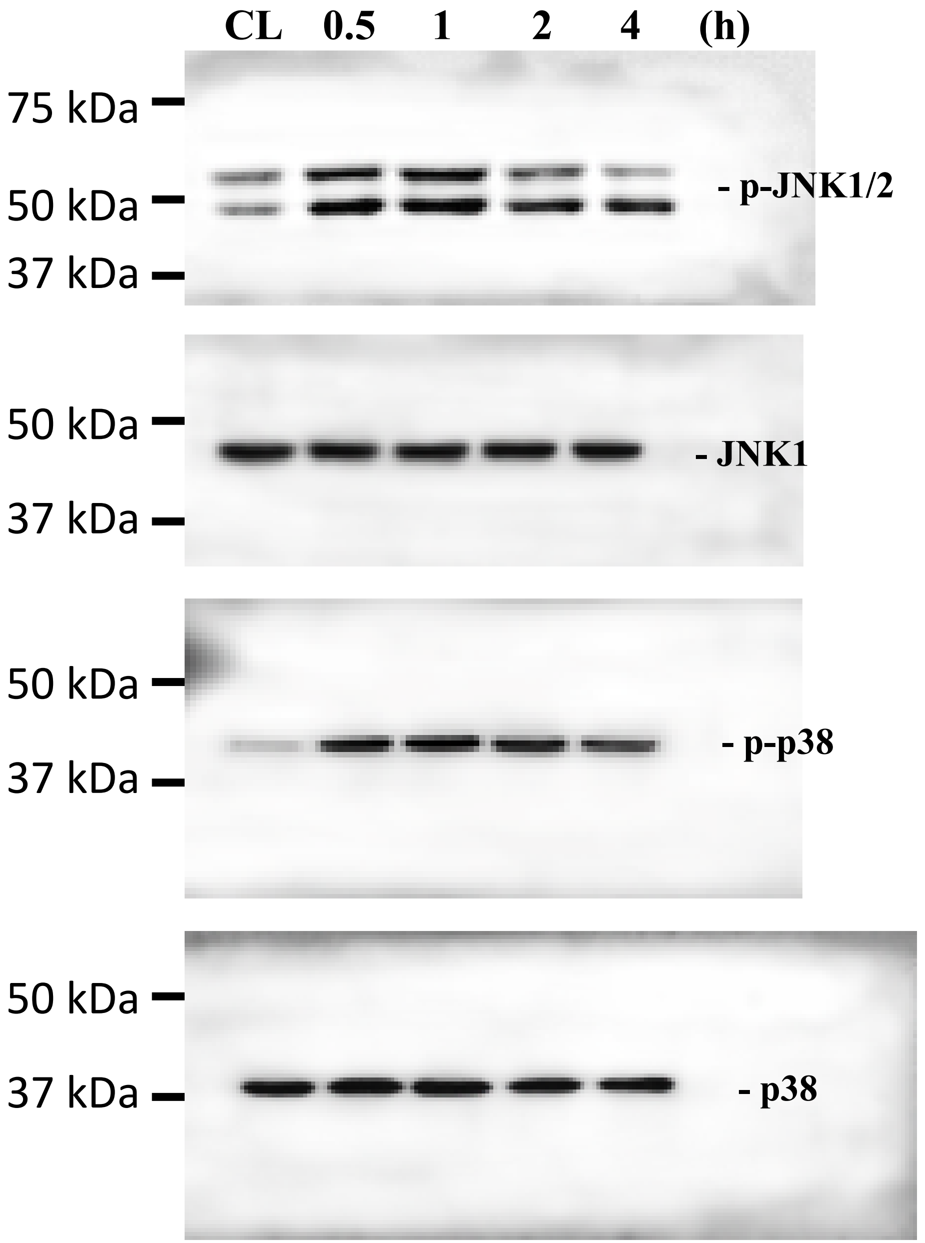

Figure S2. Raw data of Figure 3B and 3C

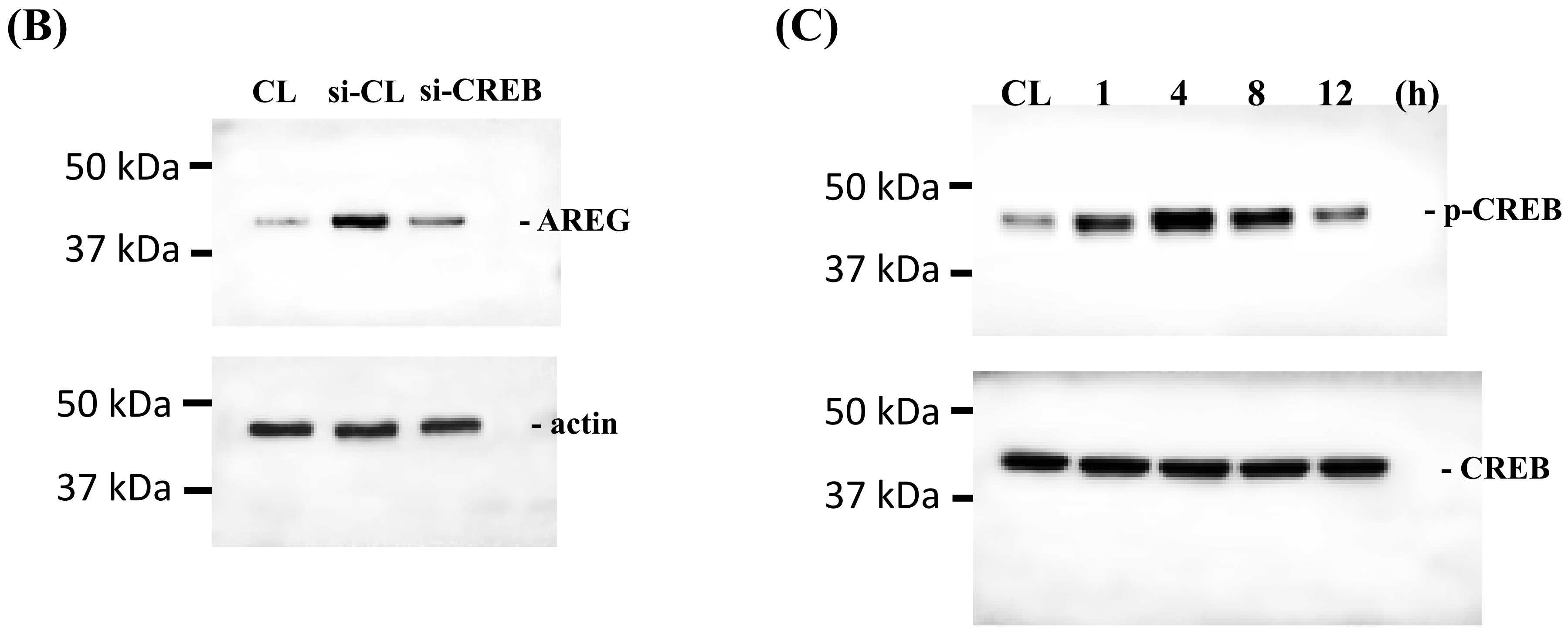

Figure S3. Raw data of Figure 4B and 4C

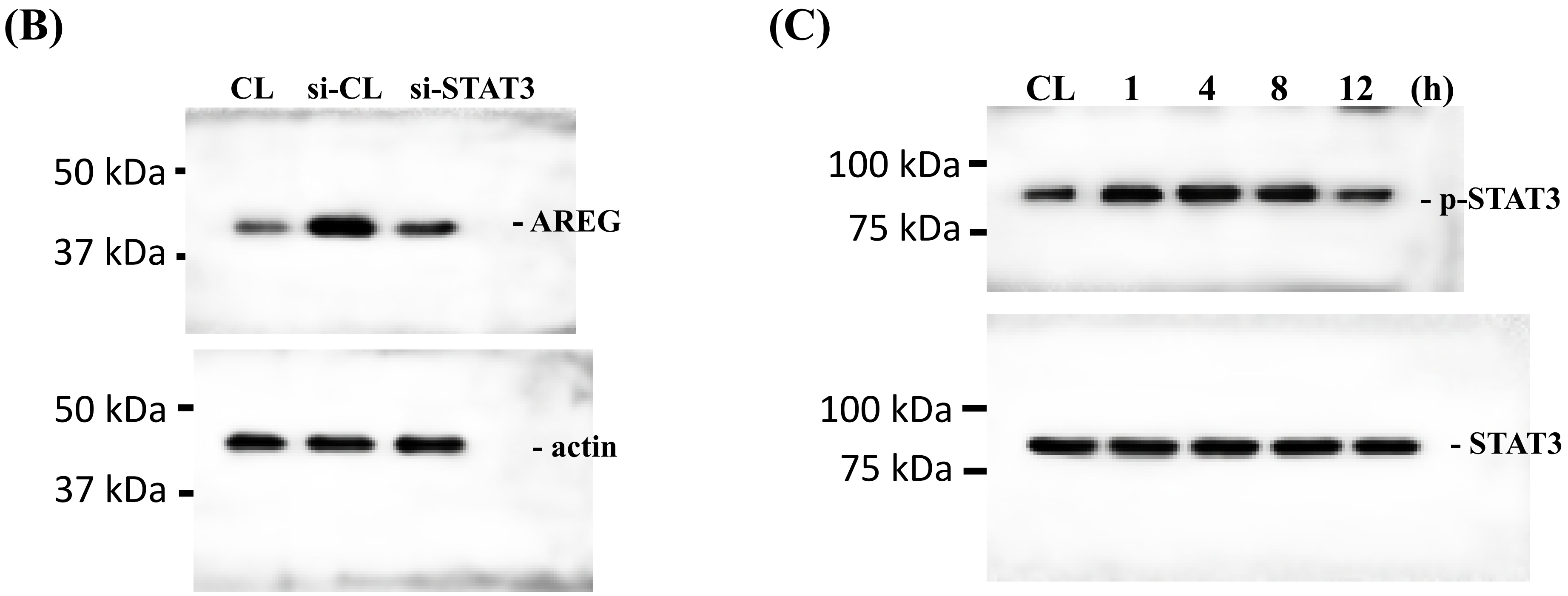

Figure S4. Raw data of Figure 5B and 5C

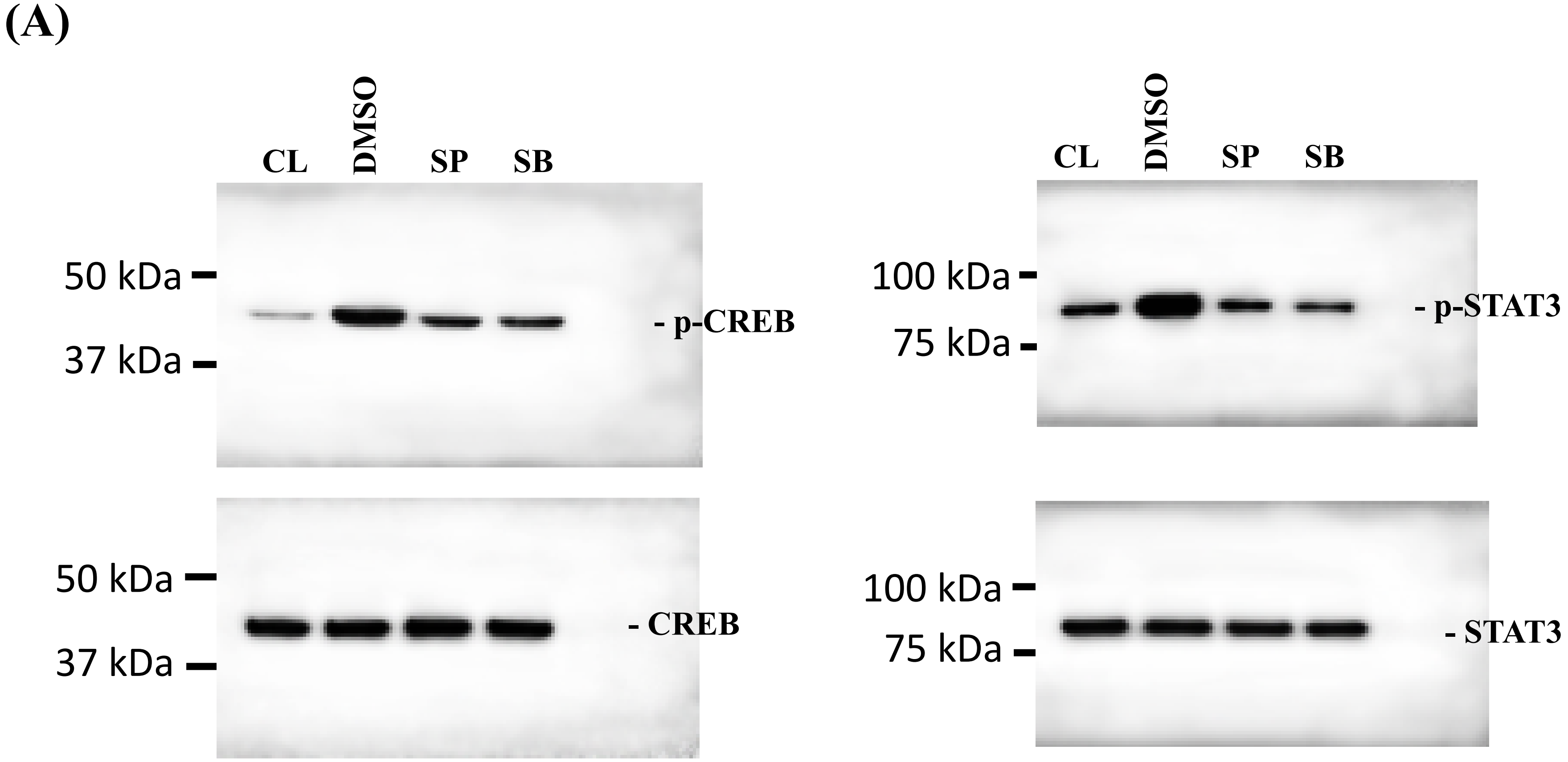

Figure S5. Raw data of Figure 6A
